# Supplementary material for: Determinants of epidemic size and the impacts of lulls in seasonal influenza virus circulation
Source: Nat Commun. 2024 Jan 18;15:591. doi: 10.1038/s41467-023-44668-z (PMC10796432; doi:10.1038/s41467-023-44668-z)
Supplement: Supplementary file 1 — Supplementary Information [file 41467_2023_44668_MOESM1_ESM.pdf]

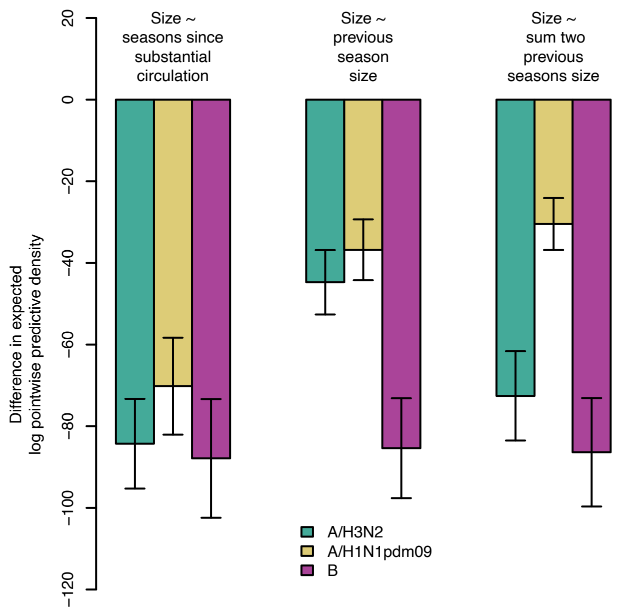

Supplementary Fig. 1. Model comparison. The difference in expected log posterior predictive density between the same models with and without season effects, for all three main model formulations discussed in the main text. Error bars indicate the standard error of the difference. ELPD quantifies the posterior predictive accuracy of a model, while penalizing model complexity. A higher ELPD means better model fit. Hence, the negative differences suggest that, for all model formulations, models that incorporate season effects have substantially superior posterior predictive accuracy compared to those that do not incorporate season effects.

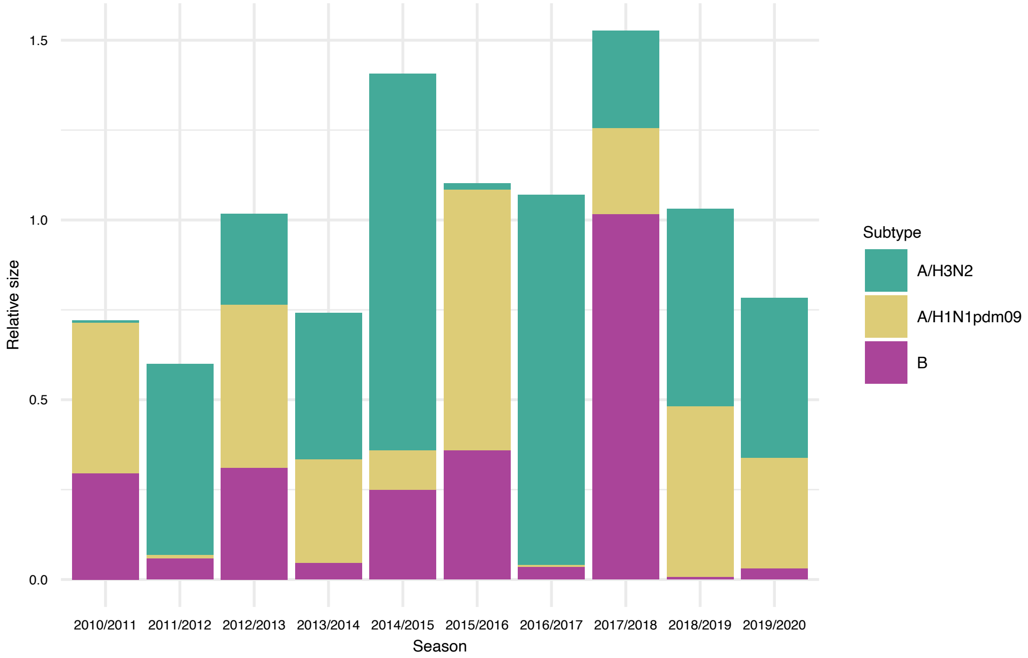

Supplementary Fig. 2. Relative sizes of seasonal influenza epidemics in the Netherlands in the decade preceding the COVID-19 pandemic. The relative size of influenza A/H3N2, A/H1N1pdm09, and B epidemics in the Netherlands, defined as the influenza-like illness rate in the Netherlands per season multiplied by the proportion of isolates corresponding to each (sub)type in each season, scaled such that a relative size of one corresponds to the mean total influenza-like illness rate in a single season.

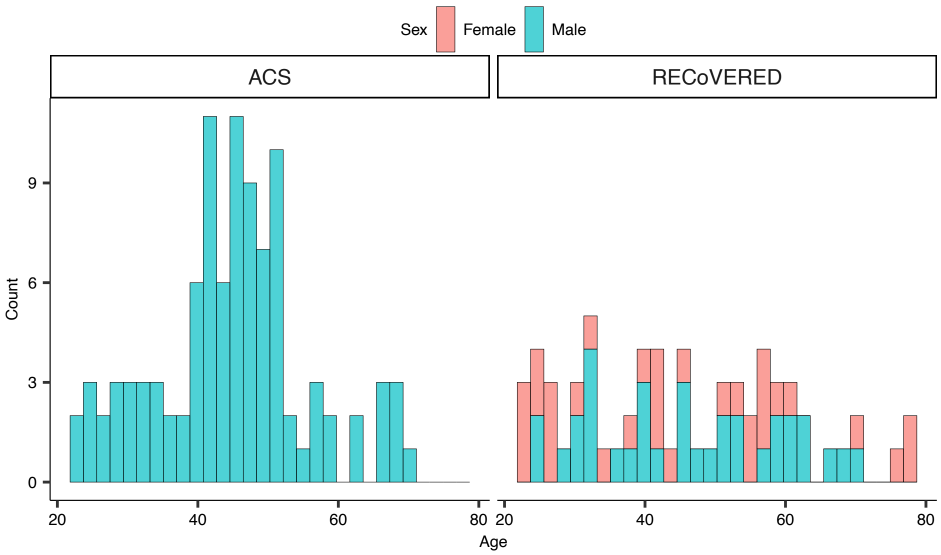

Supplementary Fig. 3. Age and sex distribution of participants in both cohorts. For the ACS and RECoVERED cohorts separately, the counts of participants, stratified by age and sex in 2021.

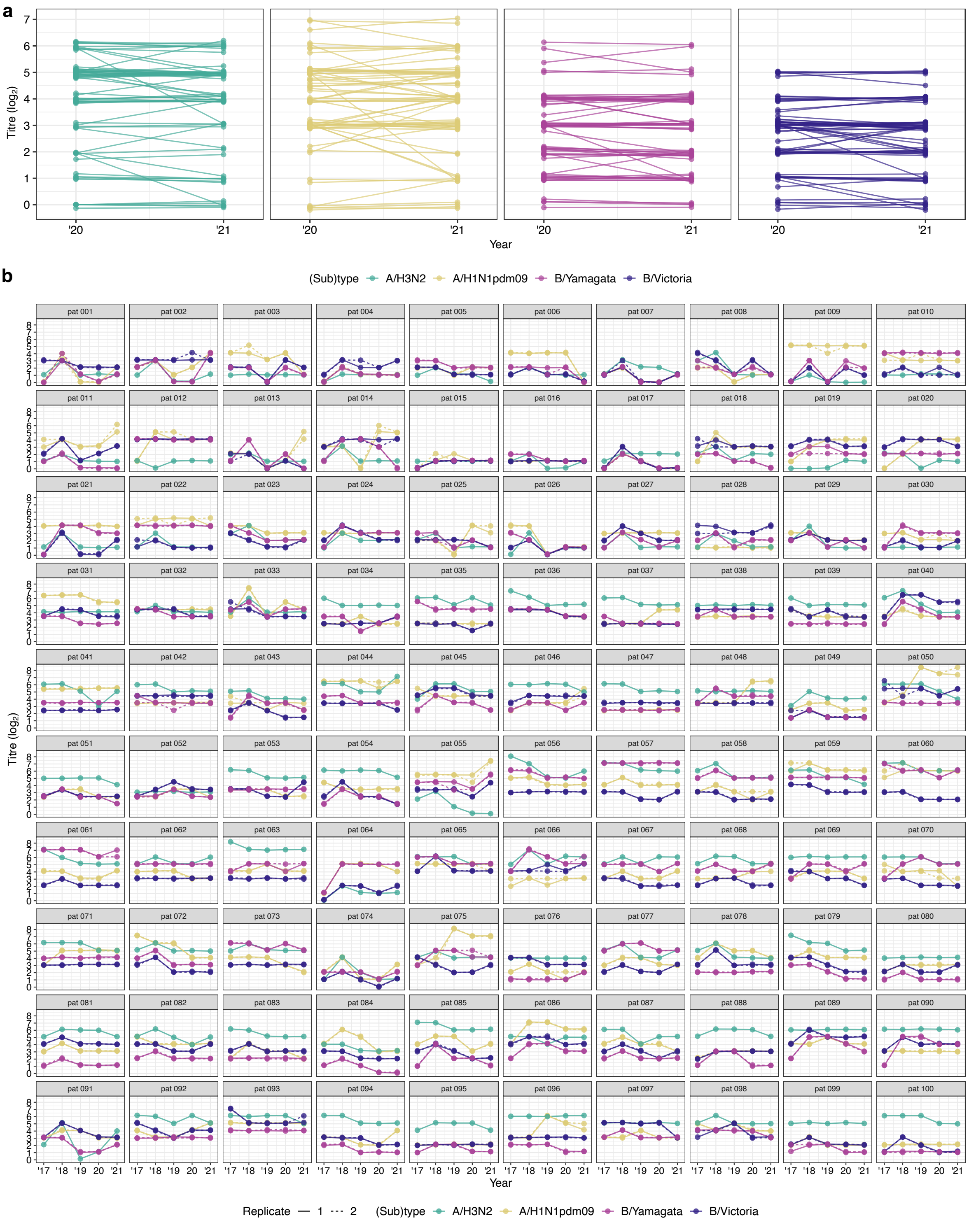

Supplementary Fig. 4. Individual HI titre dynamics for two independent cohorts.
a) (Sub)type-specific titre measurements, averaged over two replicates, for all 65 individuals within the RECoVERED cohort. Slight random jitter has been added to both panels in the *x* and *y* dimensions to avoid obscuring of repeated data points. **b)** Each panel depicts log_2 individual HI titres from 2017-2021 for all 100 participants in the Amsterdam Cohort Studies on HIV infection and AIDS (ACS). Strains are differentiated by colour and, where applicable, replicate measurements are indicated with dashed lines.

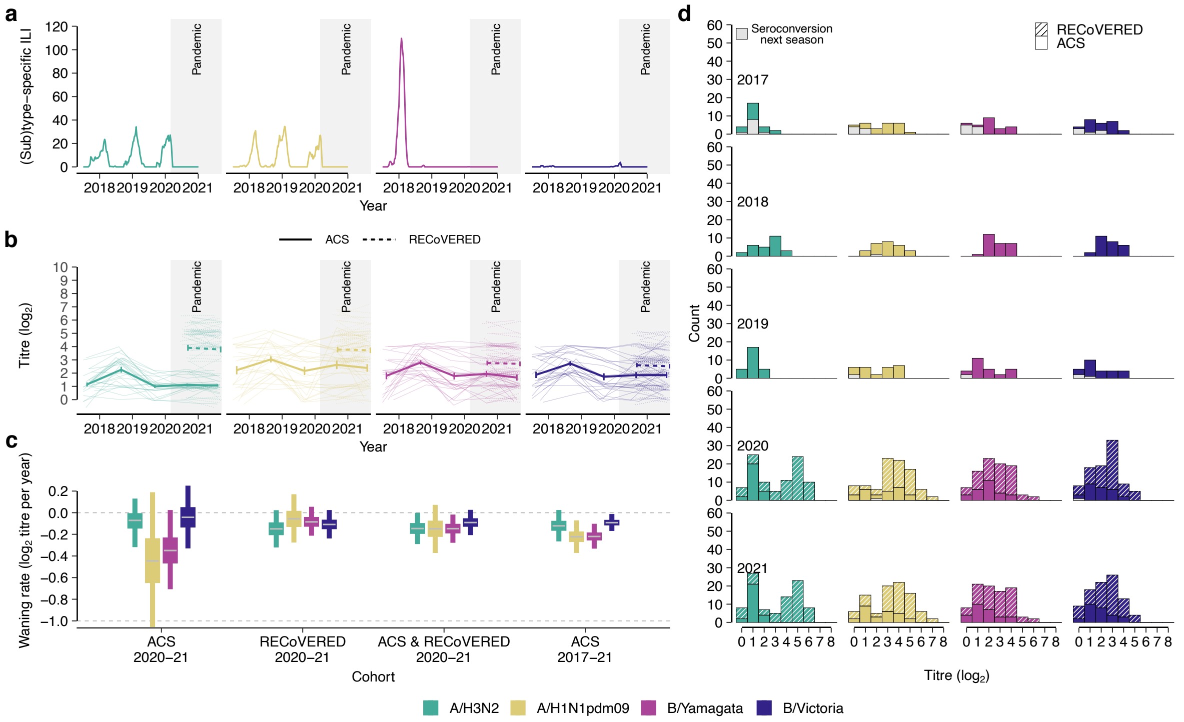

Supplementary Fig. 5. Waning antibody titres to seasonal influenza virus before and during the COVID-19 pandemic for a subset of the Amsterdam Cohort Studies on HIV infection and AIDS (ACS). The information content of this figure corresponds to that contained in Fig. 3, but for individuals 1-30 of the ACS cohort (see Methods). **a)** Seasonal influenza virus epidemic activity 2017-2021 in the Netherlands, computed as the number of reported cases of influenza-like illness per 100,000, stratified by (sub)type as estimated from virological surveillance data. **b**) Individual antibody titres against seasonal influenza viruses based on haemagglutination inhibition (HI) assay from 2017-2021 among 30 healthy male adult participants of the Amsterdam Cohort Studies on HIV infection and AIDS (ACS) cohort for each influenza virus (sub)type as well as 34 male and 31 female participants of the RECoVERED cohort for years 2020-21 (dashed). Mean antibody titre changes across all individuals are drawn in bold lines with error bars indicating the mean standard error. The viruses used are A/Netherlands/04189/2017 (A/H3N2), A/Netherlands/10218/2018 (A/H1N1pdm09), B/Netherlands/04136/2017 (B/Yamagata), and B/Netherlands/00302/2018 (B/Victoria). **c**) HI titre distributions in the two cohorts following each winter epidemic period coloured by influenza virus (sub)type. HI titre distributions of individuals who experienced a ≥2 log_2 units increase in HI titre (≥4-fold increase in HI titre) in the next winter epidemic period, indicating likely infection, are shown in grey bars. **d**) Mean HI antibody titre waning rates by influenza virus (sub)type in adults estimated from HI titres from the individuals among the 30 ACS individuals that did not see a ≥2 log_2 units increase in titre in consecutive years in the study period and 65 RECoVERED participants. Error bars correspond to the 50% and 95% credible intervals. Waning rate of -1.0 corresponds to one two-fold decrease in antibody titre in one year.

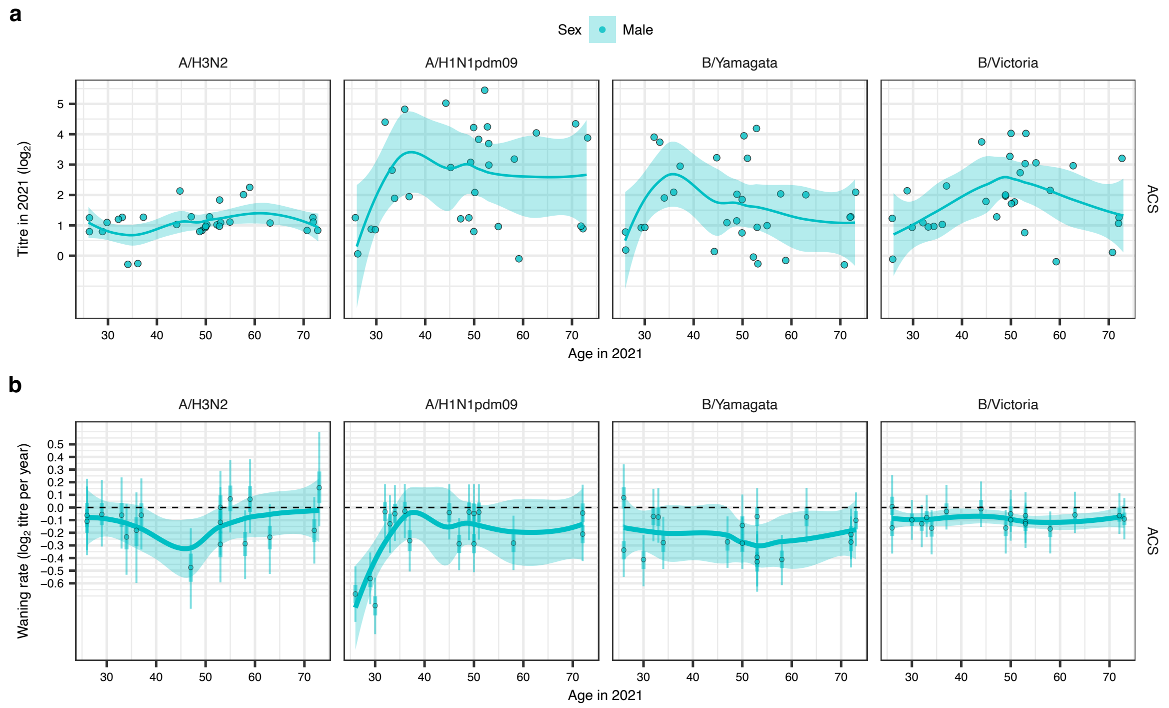

Supplementary Fig. 6. The effects of age and sex on baseline antibody titre and waning rate. The information content of this figure corresponds to that contained in Fig. 4, but for individuals 1-30 of the ACS cohort (see Methods). **a)** A cross section of antibody titres in 2021 for 30 ACS individuals, broken down by (sub)type, age and sex. The smoothing line corresponds to a LOESS fit with span = 0.75, for each sex individually. The confidence band corresponds to a 95% confidence interval. **b)** Individual-level fitted waning rates with 50% (thick lines) and 95% (narrow lines) CIs for the 2017-21 period for the individuals among the 30 ACS individuals that did not see a ≥4-fold increase in titre in consecutive years and the 65 RECoVERED individuals for 2020-21, broken down by (sub)type, age and sex. Points correspond to median individual-level fitted waning rates. The smoothing line corresponds to a LOESS fit with span = 0.75, for each sex individually. The confidence band corresponds to a 95% confidence interval.

Virological surveillance data	Influenza-like illness data
Albania	Austria
Argentina	Belgium
Australia	Croatia
Austria	Czechia
Belarus	Denmark
Belgium	Estonia
Bulgaria	Hungary
Canada	Iceland
Croatia	Ireland
Czechia	Israel
Denmark	Italy
Estonia	Latvia
Finland	Lithuania
France	Luxembourg
Germany	Netherlands
Greece	Portugal
Hungary	Serbia
Iceland	Slovakia
Iran	Spain
Ireland	Switzerland
Israel	
Italy	
Japan	
Kazakhstan	
Latvia	
Lithuania	
Luxembourg	
Netherlands	
New Zealand	
Norway	
Poland	
Portugal	
South Korea	
Romania	
Russia	
Serbia	
Slovakia	
Slovenia	
South Africa	
Spain	
Sweden	
Switzerland	
Turkey	
Ukraine	
United Kingdom	
United States of America	

Supplementary Table 1. The countries included in the analysis for the virological surveillance data and influenza-like illness data.
