# Supplementary material for: Determinants of epidemic size and the impacts of lulls in seasonal influenza virus circulation
Source: Nat Commun. 2024 Jan 18;15:591. doi: 10.1038/s41467-023-44668-z (PMC10796432; doi:10.1038/s41467-023-44668-z)
Supplement: Supplementary file 3 — Description of Additional Supplementary Files [file 41467_2023_44668_MOESM3_ESM.pdf]

Description of Additional Supplementary Files

File Name: Supplementary Data 1
Description: GISAID Acknowledgement Table
