# Supplementary material for: Determinants of epidemic size and the impacts of lulls in seasonal influenza virus circulation
Source: Nat Commun. 2024 Jan 18;15:591. doi: 10.1038/s41467-023-44668-z (PMC10796432; doi:10.1038/s41467-023-44668-z)
Supplement: Supplementary file 5 — Reporting Summary [file 41467_2023_44668_MOESM5_ESM.pdf]

nature portfolio

Corresponding author(s): C.A. Russell

Last updated by author(s): 2023/12/05

Reporting Summary

Nature Portfolio wishes to improve the reproducibility of the work that we publish. This form provides structure for consistency and transparency in reporting. For further information on Nature Portfolio policies, see our Editorial Policies and the Editorial Policy Checklist.

Statistics

For all statistical analyses, confirm that the following items are present in the figure legend, table legend, main text, or Methods section.

n/a	Confirmed	
☐	☒	The exact sample size (*n*) for each experimental group/condition, given as a discrete number and unit of measurement
☐	☒	A statement on whether measurements were taken from distinct samples or whether the same sample was measured repeatedly
☐	☒	The statistical test(s) used AND whether they are one- or two-sided<br>*Only common tests should be described solely by name; describe more complex techniques in the Methods section.*
☐	☒	A description of all covariates tested
☒	☐	A description of any assumptions or corrections, such as tests of normality and adjustment for multiple comparisons
☐	☒	A full description of the statistical parameters including central tendency (e.g. means) or other basic estimates (e.g. regression coefficient) AND variation (e.g. standard deviation) or associated estimates of uncertainty (e.g. confidence intervals)
☒	☐	For null hypothesis testing, the test statistic (e.g. *F*, *t*, *r*) with confidence intervals, effect sizes, degrees of freedom and *P* value noted<br>*Give P values as exact values whenever suitable.*
☐	☒	For Bayesian analysis, information on the choice of priors and Markov chain Monte Carlo settings
☒	☐	For hierarchical and complex designs, identification of the appropriate level for tests and full reporting of outcomes
☒	☐	Estimates of effect sizes (e.g. Cohen's *d*, Pearson's *r*), indicating how they were calculated

Our web collection on statistics for biologists contains articles on many of the points above.

Software and code

Policy information about availability of computer code

Data collection	No code or software was used to collect the haemagglutination inhibition and epidemiological data used in this study. The haemagglutination inhibition data was the outcome of the haemagglutination inhibition assay and the epidemiological data was directly downloaded from the corresponding databases as described in the data availability section.
Data analysis	Custom code was written in R (version 4.0.3) for analysis of antibody titre data and analyses of epidemiological data. Statistical modelling was performed using Stan v2.21.0. Custom scripts used for data analysis and modelling are available at the project GitHub repository (https://github.com/AMC-LAEB/waning-immunity-to-flu).

For manuscripts utilizing custom algorithms or software that are central to the research but not yet described in published literature, software must be made available to editors and reviewers. We strongly encourage code deposition in a community repository (e.g. GitHub). See the Nature Portfolio guidelines for submitting code & software for further information.

Data

Policy information about availability of data

All manuscripts must include a data availability statement. This statement should provide the following information, where applicable:

- Accession codes, unique identifiers, or web links for publicly available datasets
- A description of any restrictions on data availability
- For clinical datasets or third party data, please ensure that the statement adheres to our policy

Raw surveillance data is available from WHO FluNet (https://www.who.int/tools/flunet) and FluID (https://www.who.int/teams/global-influenza-programme/surveillance-and-monitoring/fluid). Accession codes for GISAID data are provided as a supplementary data file. Biological materials are available for study via the Amsterdam Cohort Studies on HIV infection and AIDS (ACS) and the Viro-immunological, clinical and psychosocial correlates of disease severity and long-term outcomes of infection in SARS-CoV-2 – a prospective cohort study (RECoVERED). Processed data is provided in the source data file and available at the project GitHub repository (https://github.com/AMC-LAEB/waning-immunity-to-flu). Source data are provided with this paper.

Research involving human participants, their data, or biological material

Policy information about studies with human participants or human data. See also policy information about sex, gender (identity/presentation), and sexual orientation and race, ethnicity and racism.

Reporting on sex and gender	For the analyses of antibody titre data, we make use of two cohorts (ACS and RECoVERED). All ACS individuals included in the study were male, as this cohort only consists of males. For the RECoVERED cohort, we ensured equal sampling of male (N=34) and female sex (N=31).
Reporting on race, ethnicity, or other socially relevant groupings	The cohort for antibody titre data was not categorized by race or ethnicity.
Population characteristics	A total of 630 serum samples from 165 healthy male and female adults, including people >70 years of age (elderly), were collected in the Netherlands, longitudinally, before and during the COVID-19 pandemic in two separate cohorts: 1. the Viro-immunological, clinical and psychosocial correlates of disease severity and long-term outcomes of infection in SARS-CoV-2 – a prospective cohort study24 (RECoVERED) and 2. Amsterdam Cohort Studies on HIV infection and AIDS25 (ACS).<br>The aim of the RECoVERED cohort study is to describe the immunological, clinical and psychosocial sequelae of a SARS-CoV-2 infection. Individuals aged 16 to 85 years with laboratory-confirmed SARS-CoV-2 infection were enrolled from May 2020 until the end of June 2021 in the municipal region of Amsterdam, the Netherlands. All participants provided written informed consent. The RECoVERED study was approved by the medical ethical review board of the Amsterdam University Medical Centre (NL73759.018.20). From the RECoVERED study, we selected a total of 34 male and 31 female adults ranging from 20 to 77 years old at the time of sample collection in mid-2020, all of which had a confirmed SARS-CoV-2 infection but were otherwise healthy and unvaccinated for influenza in 2020. For these 65 individuals, samples were collected in the summer period of 2020 and 2021 only (two total for each participant).
Recruitment	The Amsterdam Cohort Studies on HIV infection and AIDS: Recruitment of volunteers started in 1984 and was performed by the Municipal Health Service Amsterdam. Recruited volunteers were all men who had sex with men in the six months prior to recruitment and lived mainly around the city of Amsterdam, The Netherlands. Recruitment was limited to men in the age range of 18 to 30 years old and was done through outreach activities at MSM meeting places, online advertisement, and participants recruiting other participants.<br><br>Viro-immunological, clinical and psychosocial correlates of disease severity and long-term outcomes of infection in SARS-CoV-2 – a prospective cohort study: Recruitment started in May 2020, non-hospitalized individuals were identified from notification data of laboratory-confirmed (polymerase chain reaction (PCR) or validated antigen test) SARS-CoV-2 infection at the Public Health Service of Amsterdam (PHSA). Eligible patients were approached via telephone by trained study staff no later than 7 days after SARS-CoV-2 diagnosis. Hospitalized participants were identified from admission data and approached in the COVID-19 wards of two academic hospitals in Amsterdam, the Netherlands. In the hospitals, SARS-CoV-2 diagnosis was based on PCR and/or SARS-CoV-2-specific serology. COVID-19 patients who had been admitted to the intensive care unit (ICU) were enrolled after leaving the ICU. With the purpose of including individuals infected during the 'first wave' of the COVID-19 pandemic in the Netherlands, a limited number of hospitalized individuals were contacted after discharge up to June 30th 2020 and within three months after SARS-CoV-2 diagnosis.
Ethics oversight	The Amsterdam Cohort Studies on HIV infection and AIDS was approved by the Medical Ethics Committee of the Amsterdam University Medical Centre of the University of Amsterdam, the Netherlands (MEC 07/182). Participation in ACS is voluntary and without incentive. Written informed consent of each participant was obtained at enrolment.<br><br>The RECoVERED study was approved by the medical ethical review board of the Amsterdam University Medical Centre (NL73759.018.20). All participants provided written informed consent.

Note that full information on the approval of the study protocol must also be provided in the manuscript.

Field-specific reporting

Please select the one below that is the best fit for your research. If you are not sure, read the appropriate sections before making your selection.

☒ Life sciences ☐ Behavioural & social sciences ☐ Ecological, evolutionary & environmental sciences

For a reference copy of the document with all sections, see nature.com/documents/nr-reporting-summary-flat.pdf

Life sciences study design

All studies must disclose on these points even when the disclosure is negative.

Sample size	For the haemagglutination inhibition assay, no sample size estimation or power analysis was possible given that it was unknown the extent to which antibody titres had waned in the two years since the start of the COVID-19 pandemic. Waning rates estimated prior to the start of the COVID-19 pandemic ranged from 3.5 to 10 years, which motivated the rationale to have longitudinal samples from the ACS cohort collected in a period of five years, before and during the COVID-19 pandemic. The aim of including samples from the RECoVERED cohort was to extend our findings beyond the ACS subjects for the period within the COVID-19 pandemic, therefore all available longitudinal samples from influenza unvaccinated individuals in 2020 were included in this study. For the epidemiological analyses, no predetermined sample size was calculated and all available data was used.
Data exclusions	Regarding the haemagglutination inhibition data, we removed data points for any subjects confirmed to have received an influenza vaccine during the study period. To calculate waning rates for subjects in the ACS in the years 2017-21, we excluded strain data for individuals who saw a 4 or greater fold change in HI titer between two successive visits. This was to remove the effects of unrecorded vaccinations and/or infections. Haemagglutination inhibition data from the 30/100 ACS subjects were treated separately in the main analyses (analysis described in the extended data figures) due to supplier change in receptor destroying enzyme during the study. For the composition dataset we excluded season-country pairs with fewer than 20 positive tests or not lying in temperate zones, and for the size dataset we excluded countries that did not have influenza like illness data for the whole period from 2010-2020, did not lie in the Northern Hemisphere, did not have the shape typical of influenza epidemics, peaking in winter with little incidence outside the winter period, had substantial periods of missing data or were otherwise not well-formed.
Replication	The haemagglutination inhibition activity of all serum samples was tested in an haemagglutination inhibition assay using two replicates per sample for A/H1N1, B/Yamagata, and B/Victoria, and one single measurement for A/H3N2. Replication was not performed for A/H3N2 due to a limited supply of glycan-remodeled turkey erythrocytes. All attempts at replication were successful. Bayesian statistical models were fitted with four individual chains.
Randomization	Randomization was not relevant to the serological analyses given that the aim was not to study differences between individuals nor group of individuals. Randomization was not relevant to the epidemiological analyses because all data was used.
Blinding	Blinding was not relevant to this study given that knowing the subject number could not influence any data nor interpretation of the data generated in this study.

Reporting for specific materials, systems and methods

We require information from authors about some types of materials, experimental systems and methods used in many studies. Here, indicate whether each material, system or method listed is relevant to your study. If you are not sure if a list item applies to your research, read the appropriate section before selecting a response.

Materials & experimental systems

n/a	Involved in the study	
☒	☐	Antibodies
☐	☒	Eukaryotic cell lines
☒	☐	Palaeontology and archaeology
☒	☐	Animals and other organisms
☒	☐	Clinical data
☒	☐	Dual use research of concern
☒	☐	Plants

Methods

n/a	Involved in the study	
☒	☐	ChIP-seq
☒	☐	Flow cytometry
☒	☐	MRI-based neuroimaging

Eukaryotic cell lines

Policy information about cell lines and Sex and Gender in Research

Cell line source(s)	Madin-Darby Canine Kidney (MDCK) cells from an in-house stock, passage number >20.
Authentication	MDCK cells could not be authenticated.
Mycoplasma contamination	All cell lines tested negative for mycoplasma contamination.

Commonly misidentified lines<br>(See ICLAC register)	No commonly misidentified cell lines were used in this study.
